# Marine microfossils: Tiny archives of ocean changes through deep time

**DOI:** 10.3934/microbiol.2024030

**Published:** 2024-08-08

**Authors:** Jasenka Sremac, Marija Bošnjak, Karmen Fio Firi, Ana Šimičević, Šimun Aščić

**Affiliations:** 1 University of Zagreb, Faculty of Science, Department of Geology, Horvatovac 102B, 10000 Zagreb, Croatia; 2 Croatian Natural History Museum, Demetrova 1, 10000 Zagreb, Croatia; 3 Franka Lisice 4, 23000 Zadar, Croatia

**Keywords:** microbiota, fossilization, biostratigraphy, paleoenvironment, raw materials

## Abstract

Microorganisms have inhabited the oceans since the dawn of Earth. Some of them have organic walls and some produce mineral tests that are usually composed of carbonate minerals or silica. They can therefore be preserved with original parts during sedimentary deposition or fossilized through permineralization or carbonization processes. The most common marine fossil groups studied by micropaleontologists are cyanobacteria, coccolithophores, dinoflagellates, diatoms, silicoflagellates, radiolarians, foraminifers, red and green algae, ostracods, and pteropods. Dormant or reproductive cysts can also be used for determinations of the fossil microbiota. Microfossils can be studied in petrographic slides prepared from rocks or separated from loosely consolidated rocks by disaggregation or dissolution and wet sieving. Their presence is sometimes recognized by biomarkers. Transmitted light microscopy and reflected light stereomicroscopy are necessary for micropaleontological studies whereas scanning electronic microscopy (SEM) aids research on the tiniest fossils and reveals fine skeletal details. Microorganisms have influenced the oxygenation of water and the atmosphere, as well as Earth's carbon cycle and have contributed to the formation of sedimentary rocks. By studying microfossils, paleontologists depict the age of the rock and identify depositional environments. Such studies help us recognize periods of stress in Earth's history and understand their influence on living organisms. Biogenic rocks, made of microfossils, can be used as raw materials, such as fossil fuels, building stone, or additives for the food industry, agricultural, or cosmetic purposes.

## 1. Introduction

Earth's microbiota includes a vast variety of small living creatures (archaea, bacteria, protists, and fungi), including the first life forms on Earth, e.g., [1]. Although not always well preserved in the fossil record, they undeniably represent the basis for the sustainability of life ever since its inception. Microorganisms have changed their abundance and diversity over geological time, enabling their usage in determination of age and interpretation of paleoenvironments.

Microfossils can be studied from a small sample of sediment or rock, making them particularly suitable for research of subsurface samples from drilled cores. Marine microfossils with mineralized tests (e.g., foraminifers, ostracods, radiolarians) are particularly abundant and well known. Organic-walled remnants (e.g., dinoflagellate cysts) can also be preserved under favorable conditions. Microfossils (tests, shells, frustules, etc.) usually range between 0.001 mm and 1 mm, but micropaleontological samples may also include larger microtaxa (e.g., some foraminifera) up to several centimeters, microscopic parts/fragments of macrobiota (e.g., conodonts), as well as small representatives of marine macrofauna (e.g., pteropods) and larval stadia of various organisms. Despite the small size of microfossils, micropaleontology, as a branch of science, arose almost two centuries ago, with the pioneering research of naturalist Christian Gottfried Ehrenberg [2],[3].

The authors of this review are from Croatia, a small but geologically very diverse country, where two very different worlds joined together during the Alpine tectonic cycle as karst terrains when dominantly Paleozoic and Mesozoic limestones and dolomites full of microfossils came into contact with the Eurasian plate. A highly dynamic water mass, the Paratethys Sea, and its descendant lakes covered the Pannonian Plain during the Cenozoic Era and were cradles for fossiliferous and hydrocarbon-bearing deposits, reflecting abrupt and extensive paleoenvironmental changes. Thus, micropaleontological studies in Croatia have flourished since they were first introduced in the middle of the 20th century by Professor Vanda Kochansky-Devidé, the first female full member of the Croatian Academy of Sciences and Arts.

Although numerous expert studies on microbiota and microfossils were published considering their occurrences and applications, we felt that one general overview might help the readers to better understand the complex interactions between the microbiota and the ever-changing world.

## 2. Available material and preparation techniques

### 2.1. Field work

During field work, paleontologists search for rock samples with microfossils visible to the naked eye or with a hand lens. Samples, including less-consolidated rocks (marls, clays), are packed and appropriately identified in field bags and their geographic position (expressed by coordinates), which are duly noted in a field notebook. The usual soft sample size is between 300 and 500 g, but it can be modified according to the situation at the outcrop or planned analyses. Consolidated rock samples (in most cases limestones or dolomites) are separated and shaped by a geological hammer, taking into consideration future cutting in the laboratory. For further information see [4].

### 2.2. Preparation techniques

**Figure 1.**
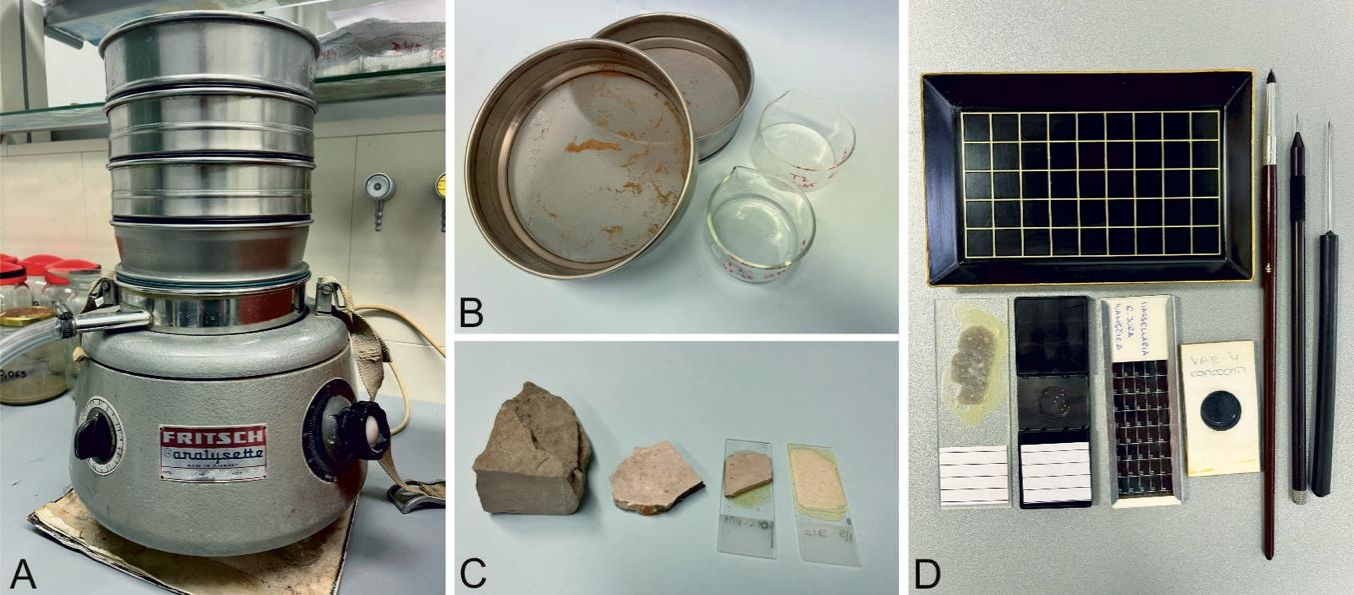
Preparation techniques in micropaleontology: Wet laboratory of the Department of Geology, Faculty of Science in Zagreb: A) Shaker for sediment sieving; B) preparation of sieved material for further analyses; C) materials representing stages in thin-section preparation–rock sample, thin slab, thin tile glued to glass slide, 20–30 micrometer-thick final thin section; and D) basic equipment for micropaleontological analysis (tray, needles and brushes, material prepared as thin sections or cells for sieved samples).

The most common preparation techniques in micropaleontology are thin sections for hard rocks and dry or wet sieving and maceration for less consolidated rocks (Figure 1). Sieving techniques, combined with the use of various acids, are used to separate the calcareous, siliceous, or phosphate exoskeletons, internal hard parts, cysts or organic-walled microfossils (palynomorphs). Sieving and fractional separation of samples improve the efficiency of paleontologists. Residual material can be further concentrated, e.g., by heavy liquids, centrifugation or electromagnetic separation ([5] and references therein).

Besides assisting in the taxonomical characterization of microbiota with simple and complex skeletal elements, thin sections are very useful for deciphering the age and depositional environment (microfacies) of the rock sample. Separation of fossils from unconsolidated sediments or loosely consolidated rocks enables study that provides data on the taxonomy and paleoecology of fossil assemblages and the age of the fossiliferous rocks (marls, clays). Such fossil associations are also suitable for morphometric and statistical analyses.

### 2.3. Photomicrographs of marine microfossils

**Figure 2.**
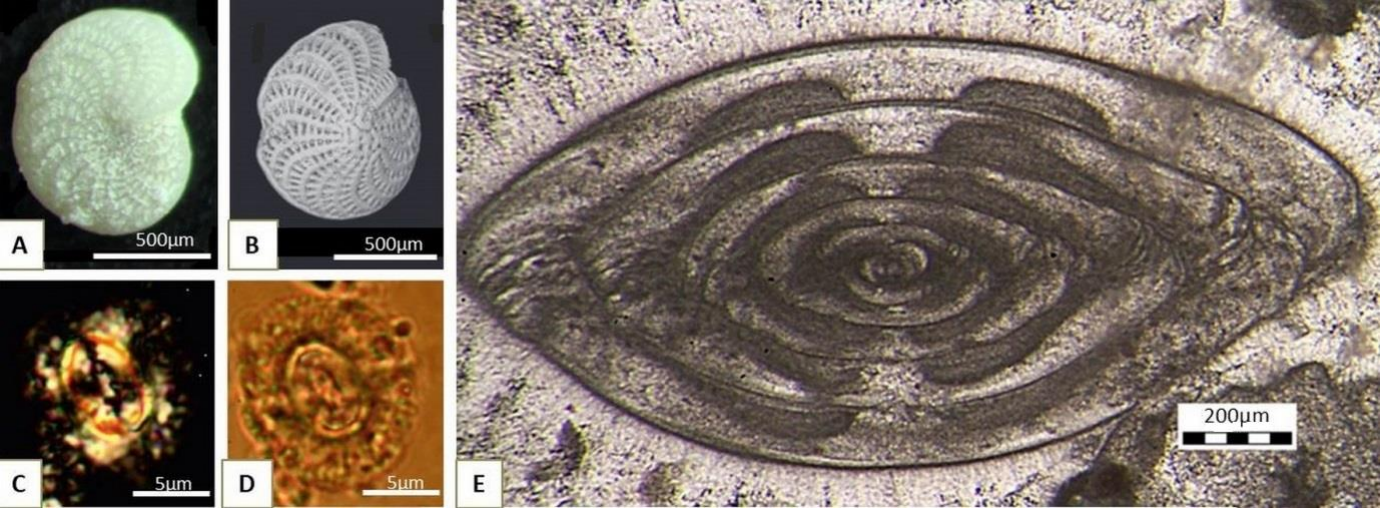
Different photomicrographic techniques: A) Foraminifera *Elphidium crispum* Linné from Sarmatian deposits of Sv. Nedelja, Croatia [6]; B) *Elphidium crispum* Linné SEM photomicrography in reflected light, Badenian deposits from Borovnjak, Croatia [7]; C) coccolithophore *Coccolithus miopelagicus* Bukry from Vejalnica, Croatia, photographed under cross polarized light (XPL); and D) under plane polarized light (PPL) [8]; E) axial cross section of the fusulinid foraminifera *Yangchienia antiqua* Kochansky-Devidé from Matkovići, Montenegro (Kochansky-Devidé coll., inv.no. 1098) photographed in a thin section using transmitted light.

A variety of photographic and digital acquisition techniques can be applied during the study of microfossils, for example, reflected-light photomicrography for microfossils extracted from unconsolidated sediments (Figure 2A). Scanning electron microscopy is highly recommended for showing minute details of the fossil (Figure 2B). Because of their very small size, coccolithophores are commonly studied with a polarizing microscope and photographed in both plane-polarized (PPL) and cross-polarized light (XPL) (Figure 2C,D). Transmitted light microscopy of thin sections is the technique of choice for relatively large benthic biota with complicated internal skeletal elements (Figure 2E).

## 3. The major groups of marine microfossils and their application

Marine microfossils comprise a variety of biota, belonging to all three domains: Bacteria and Archaea (Prokarya) have organic-walled cells that consequently exhibit a much lower preservation potential than Eukarya. Nevertheless, the Bacteria and Archaea have played a highly important role throughout Earth history, dominating Earth's biota prior to the diversification of multicellular eukaryotes some 650 million years (Ma) ago (e.g., [9],[10] and references therein).

### 3.1. Bacteria and Archaea

**Figure 3.**
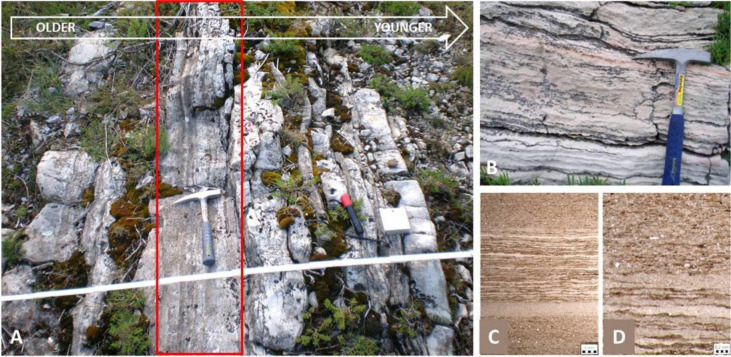
A) Upper Permian carbonate succession in the core of the Velebit anticline (Croatia), interpreted as cyclic deposition of stromatolites and bioclastic limestones in a marginal marine environment. A stratiform stromatolite horizon is under the geological hammer, marked by a red rectangle. Erosional boundary with the overlying biocalcarenite is visible at the right side of the microbialite (field photo, research [20]); B) Lower Cretaceous deposits on Mljet Island [15]; C) and D) photomicrographs of flat-lying, thinly laminated stromatolitic bedding in thin sections under transmitted light, from a bore-hole in the Sava Depression, Croatia [21].

Fossils of early prokaryotes have been reported in rocks dating back to the early Precambrian. The age of the last universal common ancestor of all living organisms, LUCA, is estimated between 4.32 and 4.52 billion years (Ga). Bacteria are the oldest known fossils, whose ancestors may date back to between 4.05 and 4.49 billion years ago, much older than the oldest known rocks at a little over 4 billion years; the estimated age of the ancestral archaea is younger, sometime between 3.37 to 3.95 billion years [11]. Though they are single-celled and small, with no mineral parts, some parts secrete extracellular polymeric substances that form biofilms that, in the case of benthic communities under certain conditions, can entrap fine sediment to form well-preserved and easily recognized laminar structures known as stromatolites and oncoids (types of microbialites) (Figure 3).

Stromatolites are the first authigenic microbial structures on Earth, dating back to 3.5 billion years or more [12]. They are most commonly attributed to the activity of cyanobacteria, which produce oxygen through photosynthesis. Hence, the expansion in the distribution of stromatolites after 2.5 Ga suggests that the cyanobacteria were responsible for one of the most important turnovers in Earth history, the Great Oxygenation Event (GOE) at 2.4 Ga [13],[14]. They are also important for paleoecological studies, as they are commonly indicative of peritidal environments (e.g., [15]) (Figure 3) and have been interpreted as crucial in the recovery of ecoystems after global catastrophes, such as the Permian–Triassic extinction (e.g., [16]–[18]). Other bacteria can secrete iron crusts or produce magnetite nanocrystals, suitable for preservation. Endolithic bacteria leave boring traces within shells, and some bacteria have even been found in amber, bone or in fossilized bone tissues [19].

### 3.2. Coccolithophores (Haptophyta Coccolithales)

Coccolithophores are phytoplanktic single-celled spherical haptophyte algae, ca. 5–100 µm across, with calcareous (low-Mg calcite), exceptionally siliceous, skeletons, known as coccospheres, composed of discoidal plates, coccoliths. All of them, except one freshwater species, live in marine environment. The oldest known coccoliths are of Late Triassic (Carnian) age, ca. 230 Ma. They are particularly flourished in the Cretaceous oceans and were strongly affected by the Cretaceous/Paleogene extinction event [22]. Today, they can be found from coastal to open ocean waters, with maximum concentrations in temperate waters [23]–[25].

Coccolithophores were first discovered by Ehrenberg (1836) in the chalk from the island of Rugen in the Baltic Sea, but Ehrenberg believed that coccoliths were of inorganic origin, even after Sorby (1861) proved the organic nature of this biota. The name coccolith was first used by Huxley (1858) [26], while the term nannoplankton, commonly applied to this group, was proposed by Lohmann [27].

**Figure 4.**
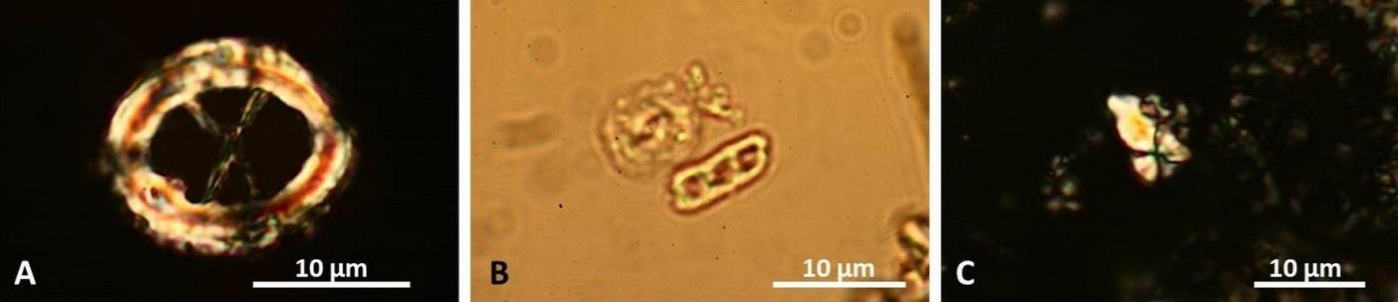
Examples of coccolith index fossils from Croatia, significant in biostratigraphy: A) *Chiasmolithus oamaruensis* from Northern Dalmatia (Promina Beds, Upper Eocene NP18 Zone); B) *Istmolithus recurvus* from Northern Dalmatia (Promina Beds, Upper Eocene NP19 Zone); and C) *Sphenolithus heteromorphus* from Medvednica Mountain, Middle Miocene NN5 Zone [8].

Coccolithophores are the main source of calcareous deep-sea oozes, which, upon compaction, produce carbonate pelagic rocks, such as the British chalk. They are excellent biostratigraphic markers, especially for the Cretaceous, Paleogene and Neogene deposits. Today we know that they strongly contribute to climatic fluctuations on submillenial timescales [28],[29]. Coccoliths are excellent tool for determining the relative age of the deposits (biostratigraphy) and paleoclimatic proxies (Figure 4). Paleotemperatures can be estimated through statistical analyses of coccolith assemblages, size or abundance of some particular taxa (e.g., *Gephyrocapsa*), Mg/Ca ratio or oxygen isotope ratio and studies of unsaturated alkenone ([24] and references therein). Coccoliths can be also used to estimate paleoproductivity ([24] and references therein, [30]). In periods of high productivity, they contribute to the accumulation of organic matter in anoxic conditions at the ocean bottom, and thus to the future formation of fossil fuels.

In ancient, as well as in modern seas, coccolithophores have been a source of nutrition. Through their production of CaCO_3_, coccolithophores contribute to the climatic balance, preventing hyperproduction of CO_2_. Their mass occurrences increase the oceans' albedo and decelerate ocean heating [31]. These effects have also been identified in the geological past.

### 3.3. Dinoflagellates (Myzozoa Dinoflagellata)

Dinoflagellates are highly diverse microorganisms, appearing not only in marine, but also in brackish and freshwater environments. Some are autotrophic, while others are heterotrophic or mixotrophic. Most live as phytoplankton, but they can also inhabit the bottom as free-living, symbiotic or parasitic forms ([32] and references therein).

Dinoflagellates in the fossil record are in most cases represented by cysts, usually ranging from 15 to 100 µm in size, with a resistant wall of organic dinosporin, calcium carbonate, or, exceptionally, silica. Cysts (also known as dinocysts) are formed by the zygote during sexual reproduction and often settle at the bottom, where they are covered by sediment. Their presence may be recognized in the biogeochemical record from dinosteranes, which are the final diagenetic product of dinosterol [32],[33]. As some cysts are not preservable and some dinoflagellate taxa do not produce cysts, their fossil record is incomplete ([32] and references therein).

The name dinoflagellate comes from *dinos* (Greek), “whirling”, which describes their distinctive swimming pattern, and *flagellum* (Latin), “a whip”. The first fossil dinoflagellate forms, from the Cretaceous deposits, were described by Ehrenberg in the 1830's. The association of dinoflagellates with their cysts was problematic for a long time, until [34] clearly related them in a published paper.

The oldest fossil remnants of dinoflagellates were found in Triassic rocks, although biogeochemical evidence, as well as molecular clock studies, point to the lineage divergence in the late Precambrian (around 650 Ma) [32]. Dinoflagellate Order Gonyaulacales is represented by fossil cysts since the Triassic but is more abundant since the Late Jurassic, whereas cysts of Order Peridinales occur since the Early Jurassic but are represented most prolifically in the Late Cretaceous and Paleogene [32].

**Figure 5.**
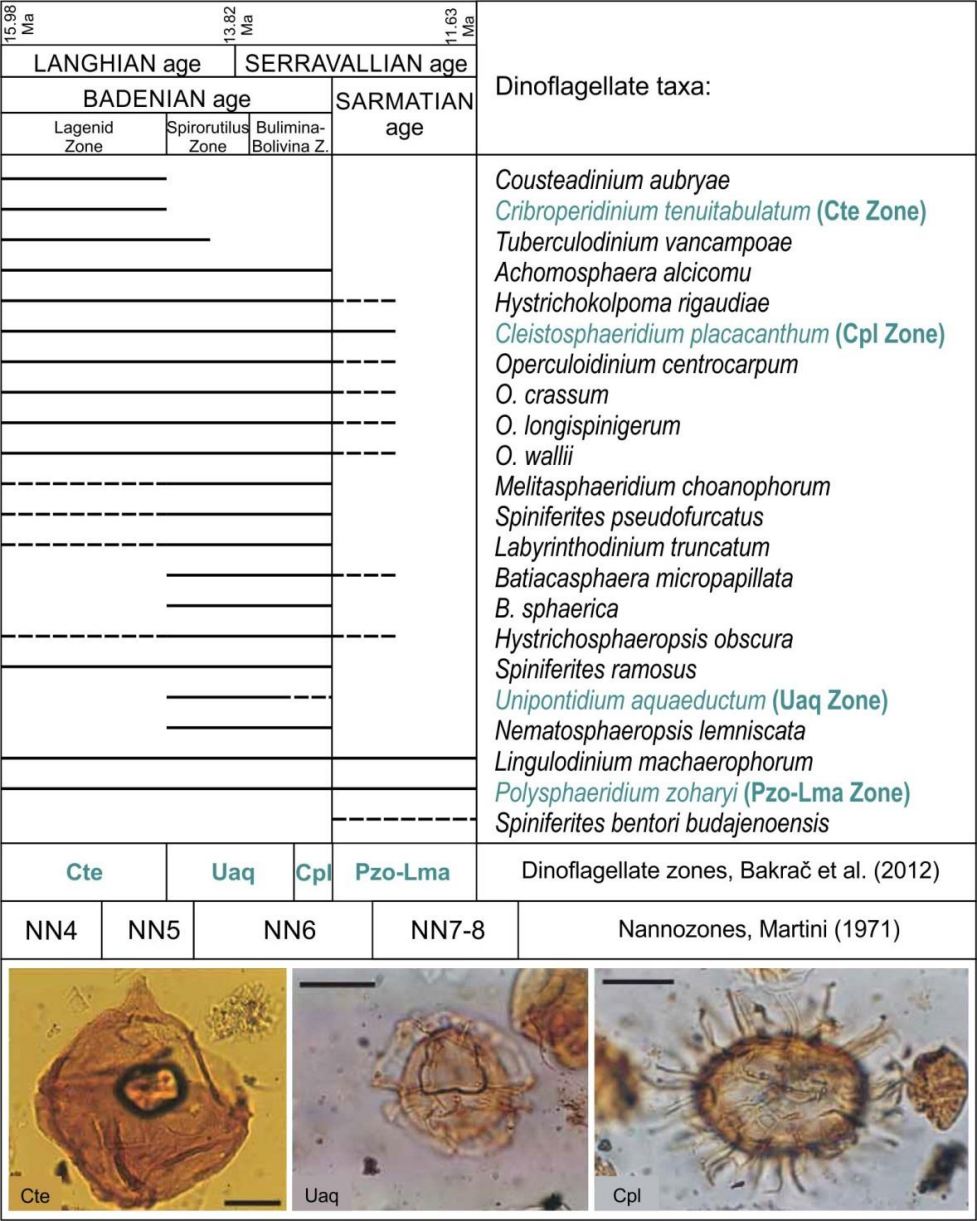
Middle Miocene marine dinoflagellate distribution and biostratigraphic zonation from Croatia based on dissertation of K. Bakrač and published data (after [36], modified), with three dinoflagellate index-fossils illustrated at bottom of figure. Names of index fossils that lend their names to the fossil zones are written in green. Note that the limits of the Cte, Uaq, and Cpl dinoflagellate zones partly differ from those of the foraminifer zones for the Badenian (at top of figure) and the nannozones (coccolithophores) at the bottom of the figure. All scales equal 25 micrometers.

Dinoflagellates can be very useful as biostratigraphic proxies. Dinoflagellate cyst stratigraphy has evolved over time, particularly since the end of the 20th century (e.g., [35]). Thus, paleontologists can use dinocysts for biostratigraphic zonation for all phases of the vast Cenozoic Paratethys Sea (e.g., [36],[37]) (Figure 5), because dinoflagellates can live not only in marine environments, but in brackish and freshwater settings as well. Specialized taxa can help in paleoenvironmental reconstructions (e.g., [38]).

Modern dinoflagellates are good environmental proxies, because they are sensitive to a variety of environmental factors, such as sea-surface temperature, salinity, and nutrient availability. A few of the modern genera have relatives way back to the Paleogene, but there are not enough paleoecological data on extinct dinoflagellates. Therefore, spatial and temporal changes in the whole assemblages are applied in paleoecological studies.

Dinocysts are commonly used as proxies for surface water productivity, by estimating the ratio between heterotrophic (peridinoid, P) and autotrophic (gonyaulacoid, G) cysts applying the formula: P/P+G. Low P/G ratio indicates low paleo-productivity, e.g., ([39] and references therein).

Additionally, organic-walled cysts can be useful in fossil fuel research, because their color can be indicative of their thermal maturity and therefore suggestive of the oil-bearing potential of the fossiliferous formation, e.g., [40].

### 3.4. Acritarchs (incertae sedis)

Acritarchs are organic-walled microfossils of uncertain taxonomical position (this is the meaning of their name, coined by Evitt, 1963), probably mostly cysts of algal affinities. They represent an artificial group of variably sculpted vesicles, varying in size between 20 and 150 µm, whose classification is rather complicated and based on their morphology.

Acritarchs occur in the Proterozoic and Paleozoic rocks, with the oldest possibly as old as 1600 to 1900 Ma. They declined since the Devonian but they can still be found in Carboniferous and Permian rocks [41].

The group comprises a valuable biostratigraphic and paleoenvironmental tool for the Proterozoic Eon and part of the Paleozoic Era, where other fossils are often lacking [41].

### 3.5. Diatoms (Gyrista Bacillariophyceae)

**Figure 6.**
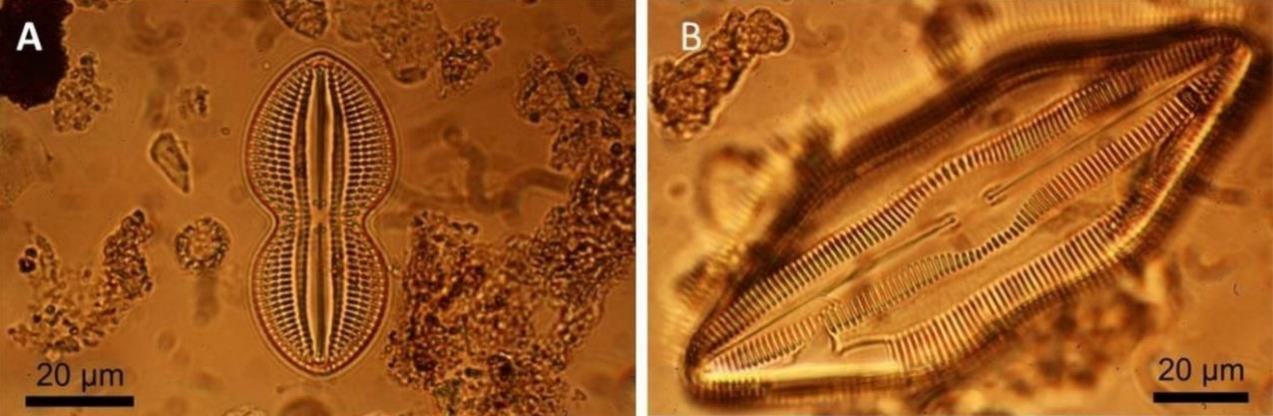
Diatoms from surface sediment in the Adriatic Sea, near the Island of Jabuka (unpublished, collected during the study for [43]). The study was applied in order to determine the age and provenance of the marine mud and sand.

Diatoms (Figure 6) are single-celled photosynthetic organisms with cell walls of transparent silica. They are present in oceans, seas, lakes, rivers, and moist soil. Today, they produce 20–30% of the oxygen we breathe and remove carbon dioxide from the atmosphere. As the most diverse group of protists on Earth, they comprise the base of the food chain in various aquatic environments. Many of them are a part of the phytoplankton, although some taxa live as the motile benthos. The size of cells ranges from 2 to 500 µm [42].

Marine planktic diatoms are among the most important contributors to phytoplankton blooms and responsible for up to 43% of marine net primary production. Their ecological success has been attributed to their ability to rapidly respond to changing environmental conditions [44].

Recent diatoms were first observed by Charles King in 1703, but they were first considered to be animals, due to their motility (e.g., Müller, 1783 from [45]). Kutzing (1844) was the first to assign them to algae.

Some doubts exist concerning the oldest body fossil of diatoms. Molecular clock estimates point to their appearance at the Triassic–Jurassic boundary (ca. 200 Ma ago), but the oldest confirmed fossils are of Early Cretaceous age [46]. The majority of diatom fossils are derived from Eocene and Miocene rocks.

Opaline diatom frustules (cell walls) can be preserved over long periods of time, without diagenetic changes. They appear in mixed assemblages, as well as in the pure siliceous oozes, even at depths over 5000 m, where all carbonate skeletal particles will be dissolved.

Most of the diatom fossil record is linked to diatomaceous earth, or diatomite [47], which has many uses in commercial exploitation (in building industry, agriculture, cosmetic industry, production of explosives). Diatomite provides an excellent base for paleoenvironmental and biostratigraphical research (e.g., [48],[49]).

### 3.6. Chitinozoans (incertae sedis)

Chitinozoans represent an unclassified group of flask-, tubular, and ball-shaped microfossils with organic-walled tests from 50 to 2000 µm in size. They have no living relatives, but there are some indications that they might be related to graptolites (Jenkins, 1970; from [41]). Their name was given by Eisenack in 1931, due to chitinous nature of the wall material.

They first appear in the early Ordovician and were abundant in the Silurian, periods for which they provide very important biostratigraphic markers. They disappear in the earliest Carboniferous [41].

### 3.7. Radiolarians (Protozoa Radiozoa) and silicoflagellates (Chromista: Silicoflagellata)

Radiolarians, a highly diverse group of unicellular holoplanktonic protozoans, produce transparent opal tests, mostly of conical, spherical, or helmet-shaped forms, from 30 to 2000 µm. They occur in rocks ever since the Cambrian and are most abundant in equatorial latitudes [50].

**Figure 7.**
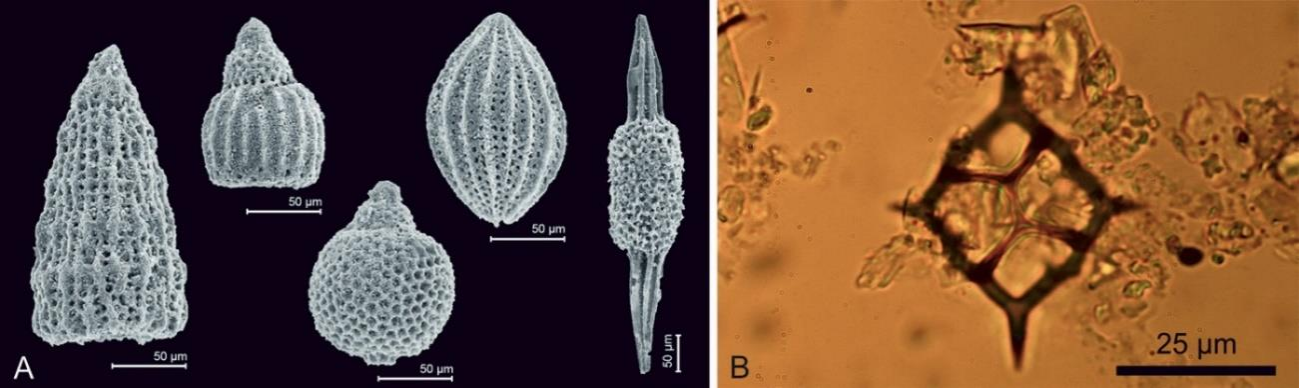
A) Oxfordian (Late Jurassic) radiolarians extracted from chert, Ivanščica Mountain, image courtesy by Duje Kukoč (collected for the study [52]); B) Silicoflagellate from subrecent sediment off the western coast of Istria, Croatia (collected during the [53] thesis research).

Ever since the beginning of the Paleozoic era, and until the rise of diatoms, radiolarians were the main constituents of siliceous oozes in deep oceans. In Mesozoic and Cenozoic rocks, as well as in modern siliceous oozes, radiolarians (Figure 7A) occur together with diatoms (Figure 6), or with less abundant silicoflagellates (Figure 7B). Similar to diatoms, they play an important role in Earth's biogeochemical cycles of silica, carbon and strontium sulfate [51]. Upon compaction, radiolarian oozes form cherts.

Radiolarians can be noted in thin sections, but for biostratigraphic research, they are studied by SEM to reveal their complex skeletons (Figure 7A, e.g., [52]).

Radiolarites, deposits composed of radiolarians, are very hard rocks which were extensively used in prehistoric production of axes, blades, drills, and scrapers, popularly named “iron of the Paleolithic” (e.g., [54]).

Silicoflagellates are marine planktic organisms with cells usually sized less than 100 µm. Their ring-like to complex dome-shaped skeletons (Figure 7B) are composed of siliceous rods. In modern seas they live in association with blue-green algae [55]. They were first noticed by Ehrenberg (1837), but Lemmermann (1901) was the first to recognize them as a separate group [56].

Silicoflagellates are known since the Oligocene and represent very useful biostratigraphical tools [57]. In Croatia they are particularly common in the Sarmatian deposits [58]–[60].

### 3.8. Foraminifera (Protozoa Foraminiferida)

**Figure 8.**
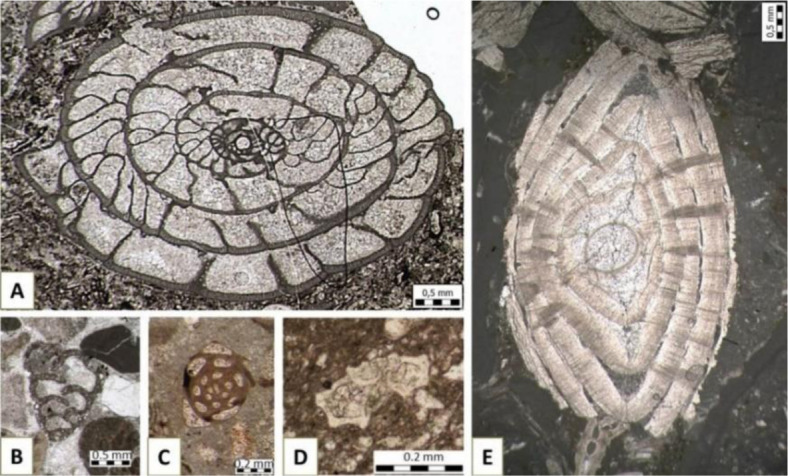
Fossil foraminifera with various types of tests in thin sections: A) *Pseudoschwagerina* (Fusulinida: Triticitidae) with microgranular calcite test from Velebit Mountain, Kochansky-Devidé collection, inv.no. 151; B) *Textularia* (Textulariida: Textulariidae) with agglutinated test, from a Miocene pebble in Quaternary gravels near Zagreb; C) miliolid foraminifera with porcellaneous test, from the Eocene of Omiš; D) planktic foraminifera *Globotruncana bulloides* Vogler (Rotaliida: Globotruncanidae) with hyaline test, Upper Cretaceous, Medvedgrad near Zagreb; and E) *Nummulites* (Rotaliida: Nummulitidae) with hyaline test, Eocene, Omiš (archives for the study [62]).

Foraminifera (Latin for the “hole bearers”) are among the most common microorganisms present in the fossil record since the Cambrian. Their tests are commonly made of calcite, but can also be of aragonite, agglutinated particles, chitin, or most rarely of silica. Tests are highly variable in shape, size, number of chambers and chemical composition, ranging from several micrometers to more than 5 cm. Foraminifera can be studied from sieved material, in reflected light, or in thin sections to observe internal skeletal elements (Figure 8).

**Figure 9.**
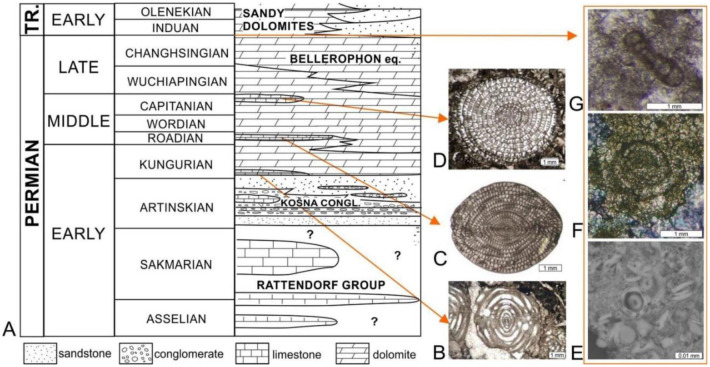
A) Modified schematic stratigraphic column through the Permian deposits of Velebit Mountain, published by [68] and modified by [69]. Three limestone zones can be easily distinguished in the field within the dominating dolomites. They comprise well preserved microfossils, particularly fusulinaceans, which can be correlated with fusulinid zones from the official Timescale [70]; B–D) fusulinid zone fossils from the Velebit Mountain: B) first limestone zone–*Eoverbeekina* from the Kochansky-Devidé collection, inv.no. 161; C) second limestone zone–*Neoschwagerina* from the Kochansky-Devidé collection, inv.no. 1963; D) third limestone zone–*Yabeina*, Kochansky-Devidé collection, inv.no. 35a; E–G) Survivor forms of foraminifera connected with the stress at the Permian–Triassic Boundary: E) small sized foraminifera *Earlandia* just above the Permian–Triassic Boundary, Rizvanuša locality in Velebit Mountain area [64]; and F) and G) small sized PTB survivor foraminifera *Ammodiscus kahlori*, from Samoborsko Gorje Hills [71].

Foraminifers inhabit a variety of environments, predominantly marine, from the shore (dominantly benthic) to the open ocean (planktic taxa) [61]. In geology, they are traditional biostratigraphic tools used to determine the age of deposits (Figure 9A–D).

Some foraminifera may be so abundant to comprise the main component of the rock. An example of such a biogenic rock is “Nummulitic limestone”, which is made up of large coin-shaped benthic foraminifera of the genus *Nummulites* (Figure 8E). This type of limestone was used as the main material for construction of the Egyptian pyramids (e.g., [63]). Significant change in diversity, abundance and size of foraminifera is commonly connected to stressful events. Environmental stress and ecological collapse associated with the mass extinction at the Permian–Triassic boundary (PTB, ca. 252 Ma ago), resulted in low-diversity, and small sized, resistant biota known as the PTB survivors (Figure 9E–G) [64]–[66].

The amount and variety of species of benthic and planktic foraminifera within a sample can furnish basic insights into both recent and past marine environments. The shells (or tests) commonly allow studies of species abundance and ratios of dominant and common species [67]. Preservational quality may help determine the autochthony of the shells, especially in benthic species.

Various indices based on studies of foraminifera can be used to estimate paleodepth and paleoproductivity, such as plankton/benthos ratio [67], the modified plankton/benthos ratio [72],[73], the Hohenegger method–using known depth ranges of benthic foraminifera [74],[75], and ratio of infaunal and epifaunal benthic forms, commonly employing statistical analyses like the PAST (PAlaeontology STatistic) program [76]. Similar methods have been used [77] on Badenian deposits from Medvednica Mountain to propose water depth and paleoenvironmental conditions within the Paratethys Sea.

Well-preserved shells can be used for geochemical analyses. Study of the stable isotope composition of carbon (δ^13^C) and oxygen (δ^18^O) in foraminiferal tests enable evaluation of past environments and environmental changes, such as paleoproductivity and environmental preferences of foraminifera using carbon isotopes (e.g., [78]) and paleotemperatures and salinity changes using oxygen isotopes (e.g., [79]). Such studies have been done for the Badenian of the Paratethys Sea (e.g., [80]–[83]), including deposits from the North Croatian Basin (NCB) [84].

### 3.9. Red algae (Plantae Rhodophyta)

Red algae comprise unicellular and multicellular taxa, abundant in marine environment, with reddish colour coming from the photosynthetic pigments. Their thalli vary in size from 20 µm to 40 cm. During their evolution, since the Mesoproterozoic, rhodophytes gradually developed the ability to secrete calcium carbonate. Such calcareous red algae have a very high preservation potential and are common in the Phanerozoic fossil record. The microscopic non-calcareous red algae *Rafatazmia* and *Ramathallus* from a ca. 1.6-billion-year-old dolomite in Central India currently represent the strongest evidence for the oldest known eukaryotes [85]. Calcareous red algae have particularly thrived since the early Jurassic and contribute greatly to growth of coral reefs by overgrowing and strengthening them. They can produce unattached nodules, called rhodoliths (maerl). Growth of calcareous red algae is controlled by temperature, nutrients, and water current velocity [86]–[88].

**Figure 10.**
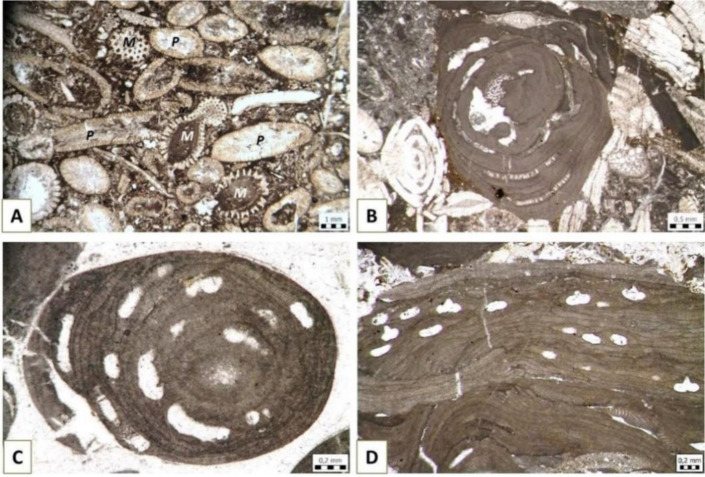
Red algae: A) Gymnnocodiacean genus *Permocalculus* (*P*) in association with green dasyclad alga *Mizzia* (*M*) in Permian deposits of Velebit Mountain, Kochansky-Devidé collection, inv.no. 1966; B) *Sporolithon*-dominated rhodolith, Eocene, Omiš, collected during the study [62],[89]; C–D) coralline algae from the Miocene “*Lithothamnion* Limestone” pebbles from the “Abesinija” gravel pit near Zagreb, collected for the study [89]; C) *Lithothamnion* sp.; and D) *Chamberlainium pentagonum* (Konti 1943) Coletti, Hrabovsky & Basso 2020.

Calcareous red algae occur in various stratigraphic formations. Particularly important for the interpretation of age and/or paleoenvironment are the Gymnocodiaceae (Figure 10A) and Corallinophycidae (Figure 10B–D).

Gymnocodiaceae are an extant group that was particularly common in Paleozoic marine shallows (e.g., [90]–[92]), while coralline algae thrive in Cenozoic intertidal and subtidal marine environments, up to 150 m deep, preferably with dim light. Recent study shows that rhodophycean bioconstructions from Eocene “Nummulitic limestones” in Dalmatia, occur as two main types. Rather regularly sphaerical, *Sporolithon*-dominated rhodoliths (unattached nodules composed dominantly of red seaweeds) (Figure 10B) were deposited on a shallow middle ramp, while complex acervulinid-rhodalgal macroids (unattached nodules made by various encrusting biota) point to deposition slightly deeper on the ramp [62],[89]. Coralline algae are also known as the important constituent of Miocene “*Lithothamnion* Limestones” [93]–[95] (Figure 10C and D), which have been mined near Zagreb as building stone for centuries, e.g., for the construction of the Zagreb cathedral [96],[97].

### 3.10. Green algae (Plantae Chlorophyta)

Green algae are highly valued among paleontologists, particularly those studying marine carbonate platform deposits. Their early fossil record is somewhat unclear, because the group does not have common, easily recognizable features, except for the pigment composition, which is usually not available for study. Fossils are common since the Ordovician. Most cells range from 2 to 7 µm in size, although the modern multi-nucleated seaweed *Caulerpa* can be up to 3 m long [98].

**Figure 11.**
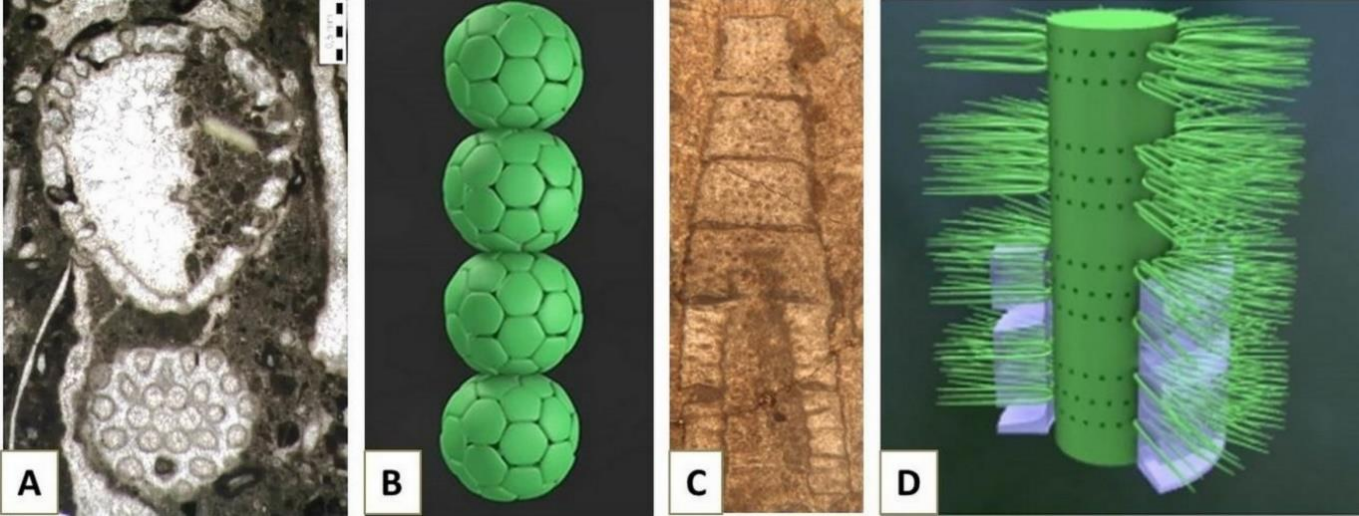
A) The calcareous alga *Mizzia velebitana* Schubert, 1908 from the Middle Permian of Velebit Mountain, Croatia, Kochansky-Devidé collection, inv.no. 41; B) model of a *Mizzia*-like alga [101]; C–D) the calcareous alga *Diplopora annulata* (Schafhäutl, 1853): C) cross section and D) reconstruction (from [102]).

Particularly important are the Dasycladales (Figure 11), inhabitants of tropical and subtropical shelves, usually up to 20 m depth. Modern dasyclad algae can grow up to 20 cm high, with even larger and far more numerous fossil representatives (nearly 200 fossil and 10 extant genera) [99]. Dasyclads secrete a tube composed of calcium carbonate, which has high preservation potential, and their remains are often so abundant, that they make up a major portion of rocks deposited on carbonate platforms. For example, most of the Dinarides, ca. 200,000 km^2^ in area with average heights of 1500–2200 m and highest peaks over 2500 m [100], are made up of rocks composed of biogenic mineral particles, in many cases those of the dasyclad algae.

Dasycladales have thrived in sheltered shallow parts of carbonate ramps since the Paleozoic, particularly during the Middle Permian (e.g., *Mizzia*, Figure 11A and B), Early Jurassic, at the Jurassic/Cretaceous boundary and during the Paleocene (e.g., [99],[103],[104]).

Some dasyclad algae, besides being paleoenvironmental proxies, are excellent index-fossils, e.g., *Diplopora annulata* (Schafhäutl, 1853), for the Ladinian (upper part of the Middle Triassic) ([104] and references therein) (Figures 11C,D).

### 3.11. Pteropods (Gastropoda Pteropoda)

Part of the abundant marine zooplankton in oceans is represented by pteropods, holoplanktic marine gastropods whose foot has been modified into wings for swimming and feeding (e.g., [105]). They can be up to 30 mm long, but most of them range from 0.3 to 10 mm in size.

Pteropoda (family Limacinidae [106]) date from the late Cretaceous (Campanian). Today, there are two recognized pteropod orders, Thecosomata (“sea butterflies”; shelled pteropods) and Gymnosomata (“sea angels”; pteropods without a shell in the adult stage). Representatives of order Thecosomata are of interest in the fossil record due to the preservation of their aragonitic shell and are divided further into the suborders Euthecosomata (shelled pteropods) and Pseudothecosomata (shelled and shell-less pteropods) (e.g., [105],[106]).

**Figure 12.**
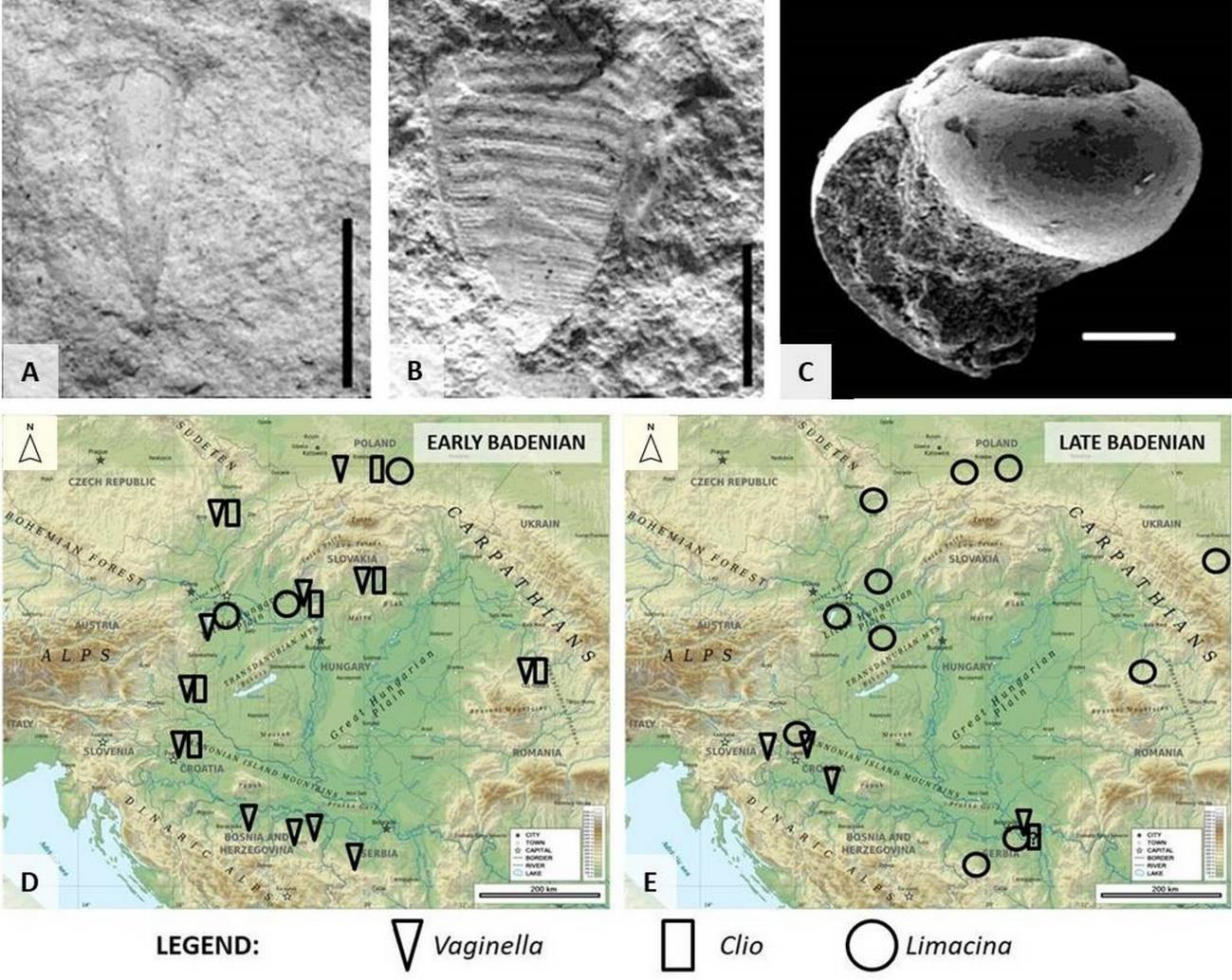
Some of the Middle Miocene pteropods from the Central Paratethys deposits and their distribution. A) *Vaginella austriaca* Kittl, 1886; B) *Clio fallauxi* (Kittl, 1886); C) *Limacina valvatina* (Reuss, 1867). Scale bars: A, B: 5 mm; C: 100 µm ([38] from [8], modified). D, E) Recorded distribution of genera *Vaginella, Clio* and *Limacina* during the early and late Badenian, respectively, at the southwestern margin of the Central Paratethys (today the Pannonian Basin and the Carpathians) [114].

Pteropod shells exhibit various morphologies, e.g. they can be sinistrally coiled, uncoiled or bilaterally symmetrical (Figure 12A–C). As one of the determining criterion for shelled pteropod species, numerical parameters, such as apical angle value or ratio between shell height and width can be used. Examples of comparisons in apical angle ranges of the Miocene *Vaginella* species (Figure 12A) are shown in e.g., ([8],[107],[108] and references therein). The usage of the shell height and width range values as the additional criterion for the Miocene *Limacina* species (Figure 12C) as shown in e.g., [109].

Different pteropod genera diversified in times of increasing seawater temperatures in different geological epochs, i.e., during the Paleocene/Eocene Thermal Maximum (PETM), the late Oligocene (Chattian), and the Early to Middle Miocene [106].

During the Early to Middle Miocene, pteropods were widespread with the rapid development of genera *Vaginella* Daudin, 1800, *Clio* Linnaeus, 1767, *Limacina* Bosc, 1817 (Figure 12A–C) and some other taxa, as evidenced by their fossil record in the Mediterranean in the “Marne a pteropodi” of northern Italy, in the Aquitaine and Carribbean basins, in the Pacific (Japan, Australia, New Zealand) [106], and the Paratethys. In Middle Miocene deposits of the Paratethys Sea, pteropods are widely distributed in the Badenian (Langhian and lower Serravallian) sediments and represented by the genera *Limacina*, *Clio* and *Vaginella* (e.g., [8] and references therein, [110]–[113]) (Figure 12).

Some of these taxa are good index fossils, e.g., *Vaginella austriaca* Kittl, 1886 or *Clio fallauxi* (Kittl, 1886). The record of pteropods in Badenian deposits of the Central Paratethys relates well to transgressive-regressive cycles and, together with other planktic biota, can be used as a tool in reconstructing migration routes during the opening of the marine corridors (e.g., [38] and references therein). Therefore, pteropods can be widely used in paleoceanographic, paleoecologic and biostratigraphic studies ([105] and references therein) (Figure 12).

Because the shell produced by pteropods is aragonitic and more sensitive to dissolution, this group of holoplanktic molluscs is today used in research evaluating the effect of ocean acidification (e.g., [115]).

### 3.12. Ostracods

**Figure 13.**
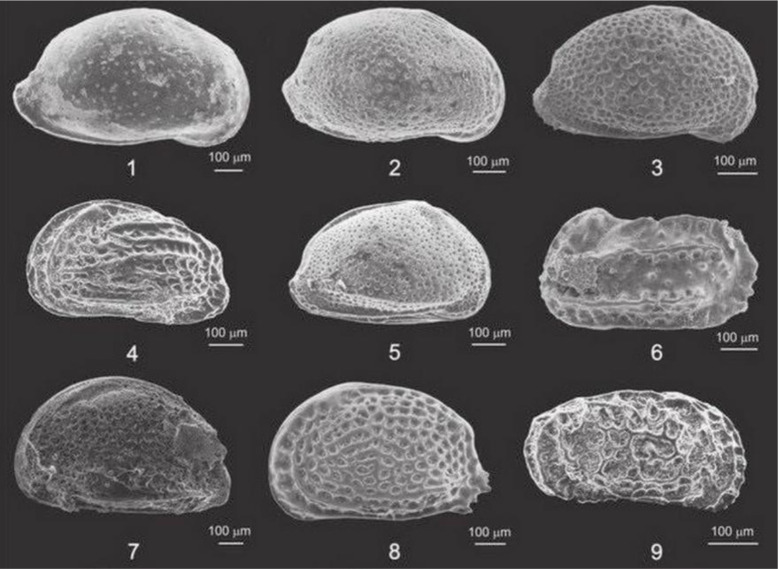
SEM photomicrographs of selected ostracods from Pokupsko and Bučica, Croatia. Ostracod assemblages from this study proved to be excellent biozonal markers and paleoenvironmental proxies for the various Middle Miocene (Badenian = Langhian, lower Serravallian) horizons. When sedimentation rates are low, valves can be well preserved and complete biocenoses can be reconstructed. Ostracod species: 1–*Aurila haueri* (Reuss); 2–*Pokonyella deformis* (Reuss); 3–*Aurila punctata* (Münster); 4–*Elofsonella* sp.; 5–*Aurila angulata* (Reuss); 6–*Carinocythereis carinata* (Roemer); 7–*Senesia* sp.; 8–*Tenedocythere sulcatopunctata* (Reuss); 9–*Callistocythere* aff. *canaliculata* (Reuss) (from [117]).

Ostracods (Figure 13), whose name is derived from the Greek *óstrakon*, meaning shell or tile, are small (usually around 1 mm) aberrant crustaceans with two-valved carapaces composed of chitin or low-magnesium calcite. They evolved in ancient seas, more than 500 million years ago, and are the most common arthropods in the fossil record. With time, they conquered a variety of environments, from brackish and freshwater to terrestrial. Their distribution is primarily affected by salinity, water temperature, pH, and dissolved oxygen [116].

Research on fossil ostracods is useful for biozonation at local and regional scales. In many countries, including those touching on the Pannonian Basin, they have greatly influenced the search for crude oil and gas, because they can be used to identify potentially productive horizons (e.g., [118] and references therein).

Ostracods can be excellent paleoenvironmental proxies (e.g., overview presented by [119]), particularly during the turbulent history of the Pannonian Basin with multiple transgressive/regressive cycles. They were among the first biota to adapt to marine ingressions into the local lakes (e.g., [120]) and to indicate fully marine regimes (e.g., [117]). Oxygen isotopes from ostracod valves, as well as the Mg/Ca ratio, can be a tool to calculate paleotemperatures (e.g., [121]).

### 3.13. Vase-shaped microfossils (VSMs) (Amoebozoa Arcellinida)

Vase-shaped microfossils comprise flask- or ball-shaped tests of early eukaryotic fossils, probably testate amoebozoans, whose name was coined by Knoll & Vidal, 1980 (from [122]). Their size ranges from ca. 40 to 300 µm in length and are sometimes present in exceptional abundances. It is presumed that the original tests were organic-walled, but most are preserved as secondary mineralized casts and molds ([123] and references therein).

VSMs are found globally in the middle Neoproterozoic, Tonian, (730–800 Ma) marine rocks, but there are some findings also in the Cryogenian succession [124]. They have been considered biostratigraphic markers, particularly as they are often the only fossils found in some Tonian rocks (mid-Neoproterozoic), but their presence in younger rocks has weakened this idea.

### 3.14. Small shelly microfossils (SSFs)/Small carbonaceous fossils (SCFs)

Small shelly fossils (SSFs) are usually less than 2 mm in length and occur in the latest Ediacaran to the end of the Early Cambrian. Their name is a name of convenience with no phylogenetic significance that was coined by Matthews and V. V. Missarzhevsky 1975 [125]. They are composed of calcium phosphate, although some are also composed of silica or calcium carbonate. Some represent the entire organism, while others are only parts of larger organisms [126].

SSFs represent important moment in animal evolution, when macroscopic life with skeletons started to occur, contemporary with the increase of calcium concentration in the ocean.

The term “small carbonaceous fossils” (SCFs) is applied to fragile remnants of animals, that can only be extracted through a maceration technique. They are relatively widespread and abundant, and can potentially preserve both mineralized and non-mineralized parts of organisms [127].

### 3.15. Conodonts

Conodonts are phosphatic denticulate structures of 0.3 to 3 mm in length that represent part of the feeding apparatus in the head region of an early vertebrate. Their name was coined by a Russian scientist Pander in 1856 [128].

Conodonts are found from the earliest Paleozoic to the Late Triassic (approximately from 540 to 200 Ma ago). Some might even be older than the Cambrian and they were particularly abundant during the Ordovician and Silurian. They occur mostly in deeper marine deposits but can also be found in shallow marine areas pointing to the variable ocean niches of these animals.

One of the most important applications of conodonts is in Paleozoic and Triassic biostratigraphy, e.g., the first appearance of the conodont species *Hindeodus parvus* (Kozur and Pjatakova 1976) marks the biostratigraphic boundary between the Permian and the Triassic [129].

Conodont fossils can be also useful in hydrocarbon exploration because their Color Alteration Index (CAI) can point to the thermal maturity of the explored deposits [130].

## 4. Discussion

**Figure 14.**
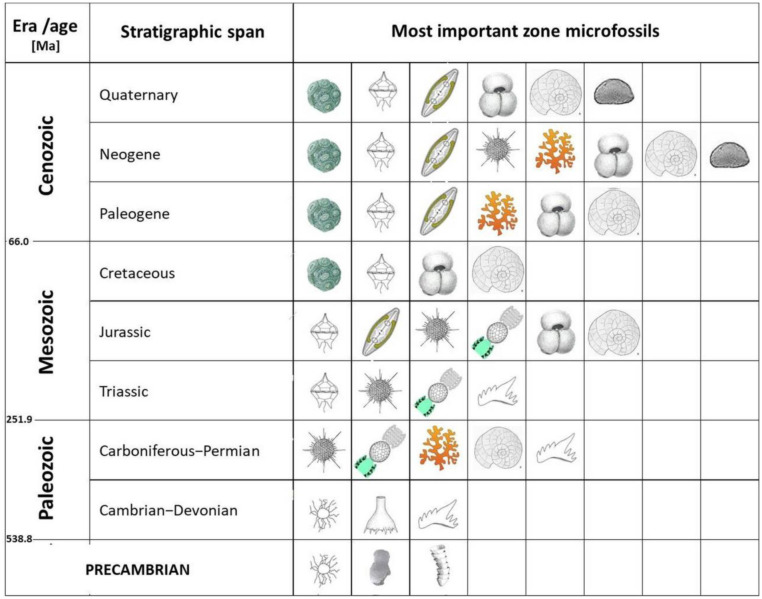
The application of microfossils in age determination through the geological periods. Legend is shown in Figure 15.

Marine microorganisms have been present on Earth since the earliest Precambrian. They are highly diverse and occur in a variety of marine environments. Their fossil record reveals only a part of their full diversity and abundance because many of them produce no hard parts and are not suitable for fossilization. Microbiota with organic-walled cells/cysts (dinoflagellates) can be fossilized in fine-grained deposits if they are quickly buried. Protozoans, some red and green algae and some mollusks (like pteropods) and crustaceans (like ostracods) produce mineral hard parts and are common in the fossil record. Nevertheless, mentioned microorganism groups are well known from the fossil record and enable us to propose relative age of studied deposits (Figure 14) or make various paleoenvironmental reconstructions.

Geologists have known for decades that microfossils can be very important biostratigraphic proxies. For example, taxa with the shortest stratigraphic span define the biozones. The most reliable results come from the combination of two or more index fossils. In the Precambrian and early Paleozoic strata only a few fossil groups can be used as biostratigraphic markers. Acritarchs are here of special importance, sometimes associated with Vase-shaped microfossils (VSMs) and Small shelly fossils (SSFs) (Figure 14). Within Cambrian, Ordovician and Silurian deposits, acritarchs and chitinozoans provide important biostratigraphic data, progressively accompanied by conodonts, which remain important all the way till the end of the Triassic (Figure 14).

Upper Paleozoic rocks comprise a variety of microfossils with rapid modes of evolution, and are thus valuable as zone fossils. Among them, fusulinid foraminifera and dasyclad algae are of highest importance for zonation of shelf deposits (Figure 9) and can be compared to the zones obtained from conodonts (Figure 14).

Since the beginning of the Mesozoic Era, some new fossil groups have taken over the main roles as biostratigraphical proxies, such as dinoflagellates and radiolarians. The bloom of the planktic foraminifera in the Jurassic makes them important zone fossils up to the present day. Coccolithophores became proliferous since the Cretaceous (Figure 14), and “nannozones”, made according to first and last appearance of certain species, make an excellent tool for dating and stratigraphic correlation between, sometimes distant, regions.

During the Paleogene and Neogene, benthic foraminifera flourish, providing the stratigraphic data for the shelf deposits. Ostracods are especially important for the realm of the ancient Paratethys Sea, known for its multiple paleoenvironmental transformations (Figure 14). For the relevant stratigraphic divisions, it is useful to compare the results obtained from the various microfossil groups with abiogenic proxies, such as the absolute age and geomagnetic data. Such complex stratigraphic divisions on the global scale are regularly updated by the International Commission on Stratigraphy.

**Figure 15.**
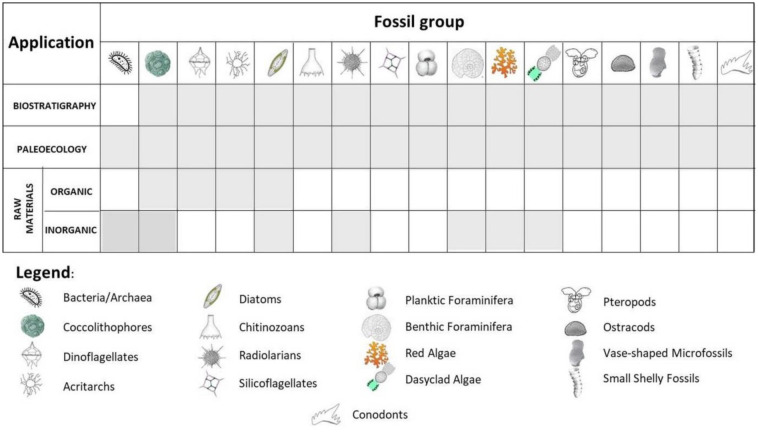
The summary of the applications of microfossils in biostratigraphy, paleoecology and the study of raw materials.

Since scientists became aware of climate changes, microfossils are given a new role as proxies of the ancient environments (Figure 15). Similar to the biostratigraphic studies, it is recommended to combine data obtained from two or more fossil groups of sensitive organisms and compare them to the sedimentological features of the studied deposits. While the biostratigraphic research pelagic biota provide more information than the bottom-dwelling taxa, sensitive benthic organisms highly depend on the bottom conditions and are commonly used as the “facies fossils” (determining the paleoenvironment). They can provide the indirect (composition of the fossil assemblage) or direct evidence (e.g., geochemical and isotope data) of ancient environmental conditions, including temperatures, salinity, oxygen saturation, and possible environmental crises of the past. While long stable periods in the past enabled development of highly diverse communities (e.g. those composed of large benthic foraminifera and dasyclad algae) (e.g., Figure 9B–D), stress periods have brought to the impoverishment of the microbiota communities, diminishing their size and leading to the survival of the small number of opportunistic taxa, which is known for the Permian/Triassic biotic crisis (Figure 9E–G).

Less known, but no less important, is the application of fossil microorganism “products” as raw materials. Some microfossils (together with associated macrofossils) make up decorative building stone (e.g., Nummulitic limestone, *Lithothamnion* limestone), or were used by early humans to produce their tools and weapons (e.g., radiolarites). Other, planktic microorganisms (e.g., coccolithophores, dinoflagellates, and diatoms) have contributed to deposition of concentrated organic matter in anoxic basin environments, thereby providing initial material for oil and gas generation. Natural chalks and diatomites have a broad palette of applications, from the cosmetic industry to agriculture, beer production and explosives.

## 5. Conclusions

Microfossils are commonly present in sedimentary rocks since the Proterozoic Eon. They can be organically preserved, preserved practically unchanged, or permineralized. Several groups of microfossils vanished through time, but many of them still have living relatives.

Some microfossil groups lived for a limited period of time and can be used to determine the age of the deposits, as well as to compare the deposits worldwide. Pelagic organisms are especially useful for the correlation among distant regions.

Taxa sensitive to environmental changes, whether benthic (more common) or planktic, can help us reveal paleoenvironmental conditions and stressful events of the past.

Fossil microorganisms are also the source of organic and inorganic raw materials.

As well as the microfossil groups that have been mentioned, their usage is even more diverse in different paleontological, geological, and climatological studies.

The development of geochemical and isotope research, optical instruments, photomicrography, and image acquisition techniques are opening new paths in micropaleontological research and will contribute to a better understanding of microbiota as tiny archives of geological history.

## Use of AI tools declaration

The authors declare they have not used Artificial Intelligence (AI) tools in the creation of this article.
